# Phytochrome B Negatively Affects Cold Tolerance by Regulating *OsDREB1* Gene Expression through Phytochrome Interacting Factor-Like Protein OsPIL16 in Rice

**DOI:** 10.3389/fpls.2016.01963

**Published:** 2016-12-26

**Authors:** Yanan He, Yaping Li, Lixin Cui, Lixia Xie, Chongke Zheng, Guanhua Zhou, Jinjun Zhou, Xianzhi Xie

**Affiliations:** ^1^Shandong Rice Research Institute, Shandong Academy of Agricultural SciencesJinan, China; ^2^College of Life Sciences, Shandong Normal UniversityJinan, China

**Keywords:** cold stress, rice, *OsDREB1*, phytochrome B, phytochrome interacting factor-like protein

## Abstract

Cross talk between light signaling and cold signaling has been elucidated in the model plant *Arabidopsis* and tomato, but little is known about their relationship in rice. Here, we report that *phytochrome B* (*phyB*) mutants exhibit improved cold tolerance compared with wild type (WT) rice (*Oryza sativa* L. cv. Nipponbare). The *phyB* mutants had a lower electrolyte leakage index and malondialdehyde concentration than the WT, suggesting that they had greater cell membrane integrity and less lipid peroxidation. Real-time PCR analysis revealed that the expression levels of *dehydration-responsive element binding protein 1* (*OsDREB1)* family genes, which functions in the cold stress response in rice, were increased in the *phyB* mutant under normal and cold stress conditions. PIFs are central players in phytochrome-mediated light signaling networks. To explore the relationship between rice PIFs and *OsDREB1* gene expression, we produced overexpression lines of rice *PIF* genes. *OsDREB1* family genes were up-regulated in *OsPIL16*-overexpression lines, which had improved cold tolerance relative to the WT. Chromatin immunoprecipitation (ChIP)-qPCR assay revealed that OsPIL16 can bind to the N-box region of *OsDREB1B* promoter. Expression pattern analyses revealed that *OsPIL16* transcripts were induced by cold stress and was significantly higher in the *phyB* mutant than in the WT. Moreover, yeast two-hybrid assay showed that OsPIL16 can bind to rice PHYB. Based on these results, we propose that phyB deficiency positively regulates *OsDREB1* expression through OsPIL16 to enhance cell membrane integrity and to reduce the malondialdehyde concentration, resulting in the improved cold tolerance of the *phyB* mutants.

## Introduction

Light is a major environmental signal influencing a multitude of steps in plant development such as seed germination, carbon assimilation, stem elongation, leaf morphology, and flowering (Carvalho et al., 2011). Plants use phytochromes to sense red light-depleted (shade) and red light-enriched (full sun) conditions (Rockwell and Lagarias, 2006). Phytochromes are light-absorbing photoreceptors that exist in two fundamental forms: the red (R)-light-absorbing form, designated Pr, and the FR-light-absorbing form, designated Pfr. The inactive Pr form is converted to the active Pfr form by exposure to R-light, and is converted back to the inactive Pr form by exposure to FR-light or through dark reversion. Phytochromes interact with members of the basic bHLH family of PIFs to regulate the expression of a large number of light-responsive genes and thus influence many photomorphogenic events (Franklin and Quail, 2010; Nagatani, 2010; Gu et al., 2011). There are five phytochrome genes (*PHYA* to *PHYE*) and eight PIF genes (*PIF1, PIF3, PIF4, PIF5, PIF6, PIF7, PIF8*, and *PIL1*) in *Arabidopsis thaliana*, and three phytochrome genes (*PHYA* to *PHYC*) and six PIL genes (*PIL11* to *PIL16*) in *Oryza sativa* (Nakamura et al., 2007; Leivar and Quail, 2011; Jeong and Choi, 2013; Luo et al., 2014).

In addition to their roles in plant photomorphogenesis, cross-talk between phytochrome-mediated light signals and cold signaling pathways has been identified in the model plant *Arabidopsis* and tomato (Williams et al., 1972; Kim et al., 2002; Wang et al., 2016). As early as 1972, Williams et al. noticed that phytochrome could mediate short day enhancement of cold acclimation, but the molecular basis for phytochrome regulation of cold tolerance remained unknown until recent years (Williams et al., 1972). CBF/DREB1 genes, which are rapidly and transiently induced by low temperatures, play an important role in cold stress responses (Gilmour et al., 2004; Heidarvand and Amiri, 2010; Mao and Chen, 2012). CBF/DREB1s can up-regulate COR genes that contain a C-repeat/drought-responsive promoter element, and thus cause metabolic changes to enhance cold tolerance (Thomashow, 2001). A low R/FR light signal increases CBF gene expression in *Arabidopsis* in a circadian clock-dependent manner. The transcript abundance of COR15a in a phytochrome-deficient mutant analysis revealed that phyB and phyD repress the CBF regulon in high R/FR light in a non-redundant manner, and the *phyD* mutant showed enhanced cold tolerance (Franklin and Whitelam, 2007). Kidokoro et al. (2009) found that PIF7 specifically binds to the G-box (CACGTG) of the *DREB1C* and *DREB1B* promoters in *Arabidopsis*. Transactivation analysis using mesophyll protoplasts revealed that PIF7 functions as a transcriptional repressor of *DREB1B* and *DREB1C* expression under circadian control, and its activity is regulated by the PIF7-interacting factors TOC1 and phyB (Kidokoro et al., 2009). Genetic analysis indicated that the CBF pathway is repressed by phyB, PIF4, and PIF7 under a warm long-day (LD) growing season, and this repression is relieved by short-day conditions. As a result, the freezing tolerance of short-day plants was increased relative to that of LD plants in *Arabidopsis* (Lee and Thomashow, 2012). Wang et al. (2016) found that FR and R light perceived by phyA and phyB positively and negatively regulated cold tolerance, respectively, in tomato (*Solanum lycopersicum*). FR light-induced activation of phyA triggers ABA signaling and, subsequently, JA signaling, leading to activation of the CBF pathway and a cold response in tomato plants (Wang et al., 2016).

Cold stress is an important factor limiting rice yield in many areas of high latitude and altitude. There are 10 putative *DREB1* homologs (*DREB1A* to *DREB1I*) in rice, six of which (*OsDREB1A, OsDREB1B, OsDREB1C, OsDREB1E, OsDREB1F*, and *OsDREB1G*) were similarly expressed in response to chilling acclimation and cold stress. Moreover, these genes were co-expressed with genes involved in cold signaling, suggesting that they might be involved in the cold response in rice (Mao and Chen, 2012). Functional analysis revealed that over-expression of *OsDREB1A, OsDREB1B*, or *OsDREB1F* in rice and over-expression of *OsDREB1D* or *OsDREB1F* in *Arabidopsis* conferred enhanced cold tolerance in transgenic plants (Dubouzet et al., 2003; Wang et al., 2008; Zhang et al., 2009). PhyB deficiency alleviated chilling-induced photoinhibition in rice, probably through a more stabilized chloroplast structure and higher unsaturated fatty acid content in membrane lipids in the *phyB* mutant (Yang et al., 2013). Cordeiro et al. (2016) observed that OsPIF14 could bind to the *OsDREB1B* promoter through two N-boxes (CACG(A/C)G) and that the flanking regions of the hexameric core were essential for protein-DNA interaction and stability. Transactivation assays using *Arabidopsis* protoplasts and rice protoplasts showed that OsPIF14 downregulates *OsDREB1B* gene expression (Cordeiro et al., 2016). Furthermore, yeast two-hybrid and Co- immunoprecipitation analyses revealed that OsPIF14 preferentially binds to the active Pfr form of rice phyB (Cordeiro et al., 2016).

In this work, we observed that *phyB* mutants exhibited enhanced cold tolerance compared with the WT, suggesting that phyB may be involved in the regulation of tolerance to cold stress in rice. To investigate the mechanism by which phyB regulates cold tolerance, we analyzed the ELI and MDA content in WT and *phyB* mutant plants. Rice plants deficient in phyB exhibited reduced ELI and MDA values, presumably as a result of increased membrane integrity. We also analyzed the expression of *DREB1* genes related to cold responses in the WT and *phyB* mutant. Genes acting upstream of the *OsDREB1* gene family were further dissected.

## Materials and Methods

### Plant Materials and Stress Treatment

*OsPIL16* overexpression, *phyB1* mutant, *phyB2* mutant and WT (*O. sativa* L., cv. Nipponbare) rice plants were used in this study. The genetic background of the *phyB1* and *phyB2* mutants was *O. sativa* cv. Nipponbare, which was described previously (Takano et al., 2001, 2005). Seeds were surface sterilized in 75% (v/v) ethanol for 30 s and then in 5% NaClO (v/v) for 20 min. The seeds were then rinsed six times in sterile double-distilled water. The sterilized seeds were soaked in water at room temperature for 2 days and then germinated for 1 day at 28°C. Seedlings were grown hydroponically in Yoshida’s culture solution as described previously (Zhou et al., 2013). Plants were cultured in a growth chamber at 28°C/28°C (day/night) under a 15-h-light/9-h-dark photoperiod. For cold stress, three-leaf stage seedlings were placed in a climatic chamber at 4°C. All treatments were repeated three times. For R light treatment, seeds from WT and *phyB* were de-husked, surface-sterilized, and grown in 0.4% agar. After incubating at 4°C overnight, the seeds were transferred to darkness for 7 days at 28°C, and then transferred to R light for another 24 h. The above-ground parts were harvested for RNA isolation.

### Cell Membrane Permeability

The cell membrane permeability of plantlets was assessed by the ELI of their tissues according to Sutinen et al. (1992) with the following modifications. The second leaves were collected from three individual plants at the three-leaf stage grown under normal conditions or treated at 4°C for different time periods. After rinsing them three times in deionized water, the samples were cut into 1 cm long sections and placed in deionized water. After the leaf sections were placed in a vacuum for 10 min and kept at room temperature for 20 min, the electrical conductivity value (named C1) was determined. Then, the samples were boiled for 30 min, and the total conductivity value (named C2) was determined after the solution was cooled to room temperature. The ELI was expressed as ELI (%) = (C1/C2) × 100.

### MDA Concentration Analysis

The MDA concentration was measured as described previously with the following modifications (Kim and Tai, 2011). The leaves were weighed and homogenized in 5 ml of 10% trichloroacetic acid solution. The homogenate was centrifuged, and 2 ml of the supernatant was added to 2 ml 0.67% thiobarbituric acid. The mixture was incubated in boiling water for 30 min, and the reaction was stopped in an ice bath. The MDA concentration was expressed as mol g^-1^ fresh mass (FM).

### Quantitative PCR

Surface-sterilized seeds of the WT and *phyB1* mutant were incubated under controlled photoperiodic conditions (15 h light, 28°C/9 h dark, 28°C) until the three-leaf stage. Samples from unstressed plants were collected as controls (0 h). Samples were taken throughout the cold stress treatment at 1, 4, 12, and 24 h after exposing the seedlings to cold stress. The collected samples were immediately flash frozen in liquid nitrogen and stored at -80°C for further analysis. Total RNA was isolated from the third leaves using the RNAiso reagent (TaKaRa, Dalian, China). First-strand cDNAs were synthesized from total RNA using a PrimeScript^TM^ RT reagent Kit with gDNA Eraser (Perfect Real Time), DNase treatment was applied before cDNAs synthesis to reduce DNA contamination according to the manufacturer’s instructions. Quantitative PCR was performed on the Thermal Cycler Dice^TM^ Real Time System (TaKaRa) using SYBR Premix Ex Taq^TM^ (TaKaRa). Each reaction contained 10 μl of SYBR Premix Ex Taq^TM^ (TaKaRa), 2 μl of cDNA sample, and 0.2 μl 20 μM gene-specific primer pairs in a final volume of 20 μl. The PCR thermal cycle used was as follows: denaturation at 95°C for 30 s and 40 cycles of 95°C for 5 s and 60°C for 30 s. As an internal control, the rice elongation factor gene (*OsEF1α*, AK061464) was used to quantify the relative transcript level of each target gene. Three biological replicates were performed. The specific primers used in this study are listed in Supplementary Table 1.

### Construction of the Plant Expression Vector and Rice Transformation

To construct the *OsPIL16*-overexpression (*OsPIL16*-OX) vector, the ORF region of *OsPIL16* was amplified by PCR from cDNA using the primers OsPIL16-F and OsPIL16-R, and cloned into the p1390-Ubi vector between the maize ubiquitin promoter and the nos terminator (Li et al., 2012). To construct the *OsPIL16*-*HA* overexpression vector, the ORF region of OsPIL16 was amplified by PCR from cDNA using the primers OsPIL16-F and OsPIL16-HA-R, and cloned in frame at its 3′ end with HA tag in the modified p1390-Ubi vector between the maize ubiquitin promoter and the nos terminator. The plasmid was introduced into *Agrobacterium tumefaciens* strain EHA105 by electroporation. Rice (*O. sativa* cv. Nipponbare) was transformed via the agroinfection method as described previously (Hood et al., 1993; Hiei et al., 1994).

### DNA Extraction and Southern Blot Analysis

Genomic DNA was isolated and purified from the *OsPIL16*-OX lines and WT plants at the six-leaf stage following Murray and Thompson (Murray and Thompson, 1980). Approximately 100 μg of DNA was digested with *Hind*III and subjected to electrophoresis on a 0.8% agarose gel. The DNA was then transferred to a nylon membrane (Hybond-N^+^; Amersham, Buckinghamshire, UK). PCR primers for amplification of the HptII gene (the selectable marker gene) are listed in Supplementary Table 1. The amplification conditions were one cycle of 3 min at 94°C, 35 cycles of 30 s at 94°C, 1 min at 56°C and 1 min at 72°C, and finally one cycle of 5 min at 72°C. The amplified fragment was purified using a PCR clean-up system. DIG-labeled probe preparation and southern blotting were performed according to the DIG High Prime DNA Labeling and Detection Starter Kit I (Roche, Mannheim, Germany) instructions.

### Yeast Two-Hybrid Assay

The full coding sequence of *OsPIF16* was amplified by PCR from cDNA using the primers OsPIL16-AD-F and OsPIL16-AD-R, and cloned into the vector pGADT7. The C-terminal non-photoactive region of *PHYB* (1891–3513 bp) was amplified by PCR from cDNA using the primers C-phyB-F and C-phyB-R, and cloned into pGBKT7. The bait and prey were cotransformed into yeast strain AH109 according to the Yeast Protocols Handbook. Then, positive yeast colonies were plated on SD -Leu -Trp -His media containing 20 mM 3-AT. pGADT7 was used as a prey for the negative control.

### *Cis*-Acting Element Analysis

The promoter sequences of *OsDREB1A, OsDREB1B, OsDREB1C, OsDREB1E, OsDREB1F*, and *OsDREB1G* were downloaded from the Rice Genome Annotation Project^1^ and used for *cis*-acting element analysis.

### Western Blot

Seedlings of WT and *OsPIL16*-*HA* overexpression lines were ground in liquid nitrogen and homogenized in a denaturing buffer (100 mM NaP_2_PO4, 10 mM Tris-Cl, and 8 M urea) by vigorous vortexing. Cell debris was removed by centrifugation at 14, 000 *g* for 10 min at 4°C. For western blot analysis, the supernatants were separated on 7.5% SDS-polyacrylamide gel, and the separated proteins were transferred to Immobilon-P-Transfer Membrane (Millipore, Bedford, USA). For detection of OsPIL16-HA, the membrane was incubated with rabbit polyclonal anti-HA antibody (Abcam, Cambridge, UK) in PBS buffer containing 0.05% Tween-20. Bands were visualized with BCIP/NBT Alkaline Phosphatase Color Development Kit (Beyotime, Nanjing, China), according to the manufacturer’s instructions.

### ChIP -qPCR Assay

ChIP assay was performed using an EpiQuik Plant Chip Kit (Epigentek, San Jose, California, USA). 14-day-old seedlings were incubated in the dark for 12 h after light irradiation and then crosslinked in 1% formaldehyde by vacuum filtration in the dark. ChIP analysis was performed by using an affinity purified anti-HA polyclonal antibody (ab9110, Abcam, Cambridge, UK) and normal IgG was used as negative control. Primers OsDREB1B-G-F and OsDREB1B-G-R, OsDREB1B-N-F and OsDREB1B-N-R that anneal to the G-box and N-box motifs in the OsDREB1B promoters, respectively, were used for qPCR. qPCR was performed using SYBR Green reagent (Takara, Dalian, Japan). Results were presented as percent of input.

All primers used in this study are listed in Supplementary Table 1.

## Results

### *phyB* Mutants Exhibit Enhanced Cold Tolerance

**FIGURE 1:**
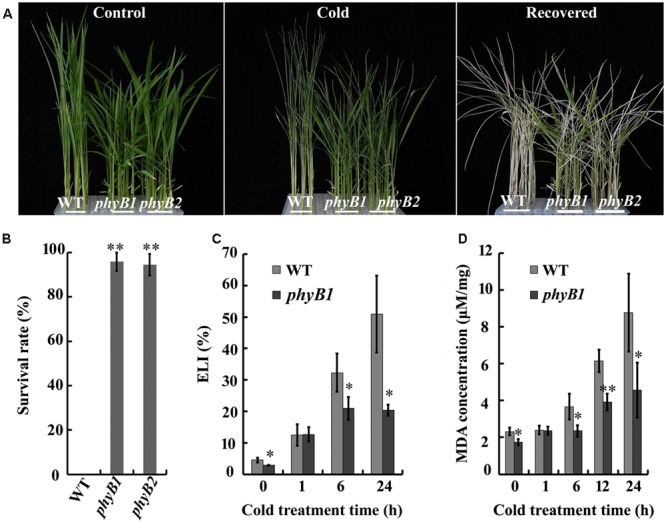
***phyB* mutants have enhanced cold tolerance. (A)** Representative photographs of wild-type (WT) and *phyB* mutants (*phyB1* and *phyB2*) under cold stress. Seedlings at the three-leaf stage were treated with cold (4°C) for 4 days and then allowed to recover at 28°C for an additional 7 days. **(B)** Survival rate of seedlings. **(C)** Electrolyte leakage index. **(D)** MDA concentration. Asterisk (^∗^) indicates that the mean value is significantly different from that of the control: ^∗^*P* < 0.05; ^∗∗^*P* < 0.01.

To test their cold tolerance, *phyB* mutant and WT rice plants were grown to the three-leaf stage in the same tray under well-watered conditions; the *phyB1* and *phyB2* mutants grew and developed normally (**Figure 1A**, Control). After 4°C treatment for 4 days, both the WT and *phyB* mutant plants showed significant wilting symptoms (**Figure 1A**, Cold). The cold-stressed seedlings were then removed to normal growth conditions for 7 days. The *phyB1* and *phyB2* plants recovered almost completely, whereas the WT plants did not (**Figure 1A**, Recovered); 95 and 94% of the *phyB1* and *phyB2* plants, respectively, survived and grew new leaves, while 0% of the WT plants grew new leaves 7 days after being removed to normal conditions (**Figure 1B**). These results unambiguously demonstrated that the phyB-deficient mutants had improved cold tolerance.

### phyB Deficiency Results in Lower ELI and MDA Concentration

To investigate the mechanism of cold tolerance, we monitored changes in the ELI, an indicator of membrane integrity. We first monitored the changes in the ELI over 24 h of cold stress in the WT and *phyB1* mutant. Under normal growth conditions, the ELI values of the WT and *phyB1* mutant were 4.5 and 2.8%, respectively (**Figure 1C**). After cold stress treatment, ELI values increased in both the *phyB1* mutant and the WT (**Figure 1C**). However, the *phyB1* mutant exhibited an obviously lower ELI at 6 and 24 h after cold treatment compared with the WT (**Figure 1C**). We speculated that phyB deficiency probably increased the ability to maintain membrane integrity under cold stress, which is likely to be one of the factors enabling the *phyB* mutants to cope with cold stress.

Malondialdehyde is the end product of lipid peroxidation that is generated from the oxidation of polyunsaturated fatty acids by ROS and other free radicals in membranes, and acts as an indicator of lipid peroxidation (Liu et al., 2013). We measured the MDA concentration in the WT and the *phyB1* mutant after cold stress treatment. The MDA concentration increased to about twofold in the *phyB1* mutant and to about fourfold in the WT after 24 h of cold stress (**Figure 1D**), consistent with the changes in ELI. The lower MDA concentration in the *phyB* mutant indicated reduced membrane lipid peroxidation relative to the WT, which probably contributed to the enhanced membrane integrity under cold stress.

### phyB Altered *OsDREB1* Expression Patterns

**FIGURE 2:**
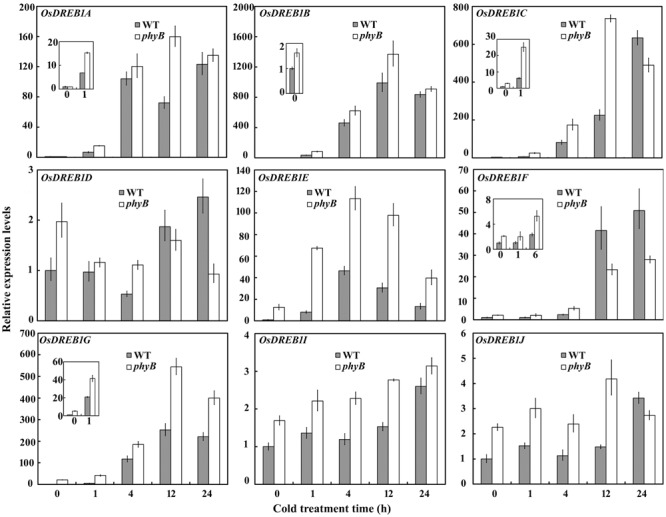
***OsDREB1* expression patterns in the WT and *phyB* mutant.** Seedlings at the three-leaf stage were treated with cold (4°C) for 0, 1, 4, 12, or 24 h. Real-time PCR was performed using cDNA derived from leaves. Error bars indicate SE (*n* = 3).

Dehydration-responsive element binding protein 1 play an important role in cold responses, as indicated by the observation that overexpression of *OsDREB1*s can enhance cold tolerance in rice (Mao and Chen, 2012). To explore whether *OsDREB1*s were involved in the enhanced cold tolerance of the *phyB* mutant, we analyzed the expression patterns of *OsDEREB1*s in WT and *phyB* mutant plants grown under normal conditions and cold stress. Six of the nine *OsDREB1* family members, including *OsDREB1A, OsDREB1B, OsDREB1C, OsDREB1E, OsDREB1F*, and *OsDREB1G*, were rapidly induced by cold stress in both the WT and *phyB* mutant, but their expression levels were relatively higher in the *phyB* mutant compared with the WT under normal conditions and cold stress (**Figure 2**). Although *OsDREB1D* was induced by cold treatment in the WT but not the *phyB* mutant, its expression was about twofold higher in the *phyB1* mutant relative to the WT under normal conditions (**Figure 2**). *OsDREB1J* and *OsDREB1I* expression was not obviously induced by cold stress, but their transcription levels were clearly higher in the *phyB* mutant than in the WT. These results demonstrated that phyB deficiency promoted *OsDREB1* family gene expression. Considering that overexpression of *OsDREB1A, OsDREB1B*, and *OsDREB1F* enhanced the cold tolerance of rice and that overexpression of *OsDREB1F* or *OsDREB1D* in *Arabidopsis* conferred enhanced cold tolerance (Ito et al., 2006; Wang et al., 2008), we speculated that increased expression of *OsDREB1* family genes probably contributed to the enhanced cold tolerance of the *phyB* mutants.

### phyB Altered the Expression Pattern of *OsPILs*

**FIGURE 3:**
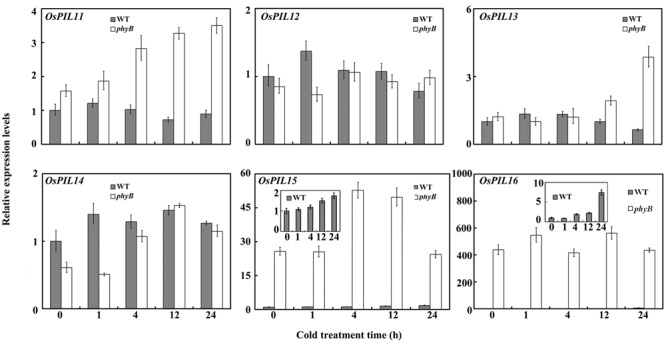
***OsPIL* expression patterns in the WT and *phyB* mutant.** Seedlings at the three-leaf stage were treated with cold (4°C) for 0, 1, 4, 12, or 24 h. Real-time PCR was performed using cDNA derived from leaves. Error bars indicate SE (*n* = 3).

How does phyB regulate *OsDREB1* expression? Because PIFs play a pivotal role in phytochrome-mediated light signaling networks (Castillon et al., 2007), we wondered whether OsPIFs were involved in the regulation of the cold response. Nakamura et al. (2007) identified six PIL homologs in rice, designated *OsPIL11* through *OsPIL16*, by extensively evaluating all of the rice databases (Nakamura et al., 2007). To dissect the role of *OsPIL*s in cold responses, we compared their expression patterns in WT and *phyB* mutant plants grown under normal conditions and cold stress. *OsPIL12* expression levels were not affected by cold stress in the WT or *phyB* mutant, while *OsPIL11, OsPIL13*, and *OsPIL14* were induced by cold stress in the *phyB* mutant but not the WT (**Figure 3**). *OsPIL15* was induced by cold stress in both the WT and *phyB* mutant, whereas cold stress induced *OsPIL16* expression only in the WT, not in the *phyB* mutant (**Figure 3**). The expression levels of *OsPIF15* and *OsPIF16* were much higher in the *phyB* mutant than in the WT under normal conditions and cold stress (**Figure 3**). In particular, *OsPIL16* transcript levels were about 400-fold higher in the *phyB* mutant than in the WT. To understand how phyB regulates *OsPIL16* gene, we compared the transcript levels between WT and *phyB* mutant seedlings that were grown in the dark, or grown in the dark but irradiated with 24 h of continuous R light before harvest. *OsPIL16* transcript level was decreased by R light in WT but not in *phyB* (Supplementary Figure 1), suggesting that phyB perceive R light to inhibit *OsPIL16* gene expression at the transcriptional level. Based on these results, we deduce that the expression of multiple *OsPIL*s was regulated by cold stress, and that phyB was involved in modulating the *OsPIL* gene expression.

### *OsPIL16*-Overexpression Lines Have Enhanced Cold Tolerance and Increased Expression Levels of *OsDREB1*s

**FIGURE 4:**
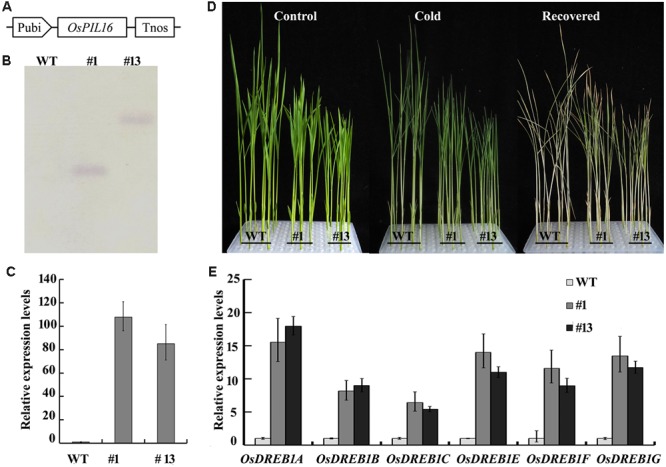
**Function of *OsPIL16* in regulating cold stress. (A)** Schematic diagram of the construct used to obtain *OsPIL16*-OX lines. The *OsPIL16* gene was driven by the maize ubiquitin promoter (Pubi). **(B)** Southern blot analysis of *OsPIL16*-OX lines. **(C)** The expression levels of *OsPIL16* in overexpression lines. Real-time PCR was performed using cDNA derived from leaves. Error bars indicate SE (*n* = 3). **(D)** Representative photographs of the WT and *OsPIL16*-OX lines under cold stress. Three-leaf stage seedlings were treated with cold (4°C) for 4 days and then allowed to recover at 28°C for an additional 7 days. **(E)** *OsDREB1* expression levels in the *OsPIL16*-OX lines and WT. Real-time PCR was performed using cDNA derived from the leaves of three-leaf stage seedlings. Error bars indicate SE (*n* = 3).

To investigate the role of *OsPIL*s in regulating cold tolerance in rice, we produced the overexpression lines of *OsPIL15* and *OsPIL16* because of the significant difference in their transcript levels between the WT and *phyB* (**Figure 3**). The *OsPIL15*-overexpression (*OsPIL15*-OX) lines were reported in our previous study (Zhou et al., 2014). In this study, we produced transgenic rice lines overexpressing *OsPIL16* (**Figure 4A**). Two independent homozygous lines of the T_4_ progeny (#1 and #13) were used to analyze the role of *OsPIL16* in rice based on their high expression levels (**Figures 4B,C**). Seedlings of the *OsPIL16*-OX lines and WT at the three-leaf stage were hydroponically grown under cold conditions for 4 days and then allowed to recover for 7 days. The *OsPIL16*-OX lines exhibited enhanced cold tolerance, as more seedlings survived compared with the WT (**Figure 4D**). However, *OsPIL15*-OX lines exhibited the same sensitivity as the WT (data not shown). These results suggested that *OsPIL16* was involved in the cold response.

In *Arabidopsis*, PIF4 and PIF7 down regulate the CBF pathway and freezing tolerance under LD conditions (Lee and Thomashow, 2012). To clarify the relationship between the OsPILs and OsDREB1s, we compared the transcript levels of the *OsDREB1*s between the WT and *OsPIL16*-OX or *OsPIL15*-OX lines. As shown in **Figure 4E**, the expression levels of *OsDREB1A, OsDREB1B, OsDREB1C, OsDREB1E, OsDREB1F*, and *OsDREB1G* were increased in the *OsPIL16*-OX lines compared with the WT. These results suggested that OsPIL16 enhanced cold tolerance by up regulating *OsDREB1* expression. However, the transcript levels of the *OsDREB1* genes were similar in the WT and OsPIL15-OX lines (Supplementary Figure 2). The expression patterns of the *OsDREB1*s were consistent with the cold tolerance of the *OsPIL16*-OX and *OsPIL15*-OX lines.

**FIGURE 5:**
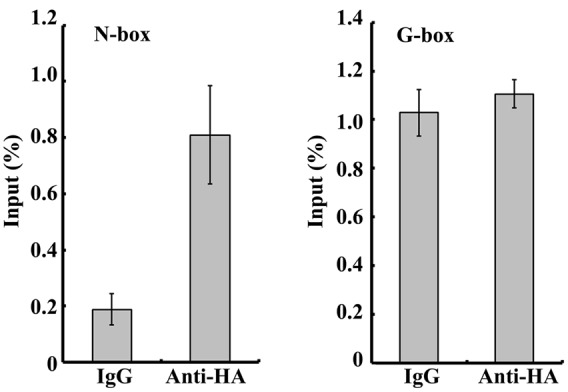
**Chromatin immunoprecipitation assays of 14-day-old seedlings grown under long-day conditions and shifted to dark for 12 h.** Relative enrichment in the G-box region and N-box region of *OsDREB1B* promoter was quantified by qPCR.

A previous study revealed that PIFs can bind to the G-box, PBE (CACATG) or N-box (CACGCG or CACGAG) (Cordeiro et al., 2016; Kim et al., 2016). Promoter analysis of *OsDREB1A, OsDREB1B, OsDREB1C, OsDREB1D, OsDREB1E, OsDREB1F*, and *OsDREB1G* revealed that they all contained a G-box, PBE, or N-box (**Table 1**). This result suggested that OsPIL16 probably up-regulates the expression of *OsDREB1* genes by directly binding to their promoter regions to enhance cold tolerance. To test this hypothesis, we produced the *OsPIL16*-*HA* overexpression (*OsPIL16*-*HA* OX) lines and detected OsPIL16-HA protein using western blotting (Supplementary Figure 3). ChIP-qPCR assays using primers that anneal to the G-box and N-box motifs in the *OsDREB1B* promoters were performed. The results show that N-box regions were enriched in the chromatin fractions by anti-HA antibody compared to that by control IgG (**Figure 5**).

**Table 1:** Promoter analysis of *OsDREB1s*.

Gene	*Cis*-acting element	Position	Sequence
*OsDREB1A*	G-box	–563 to –558	catgctccCACGTGccatagat
	N-box	–1551 to –1546	tcgcggagCACGCGgtgttgtg
*OsDREB1B*	G-box	–2896 to –2891	tatcaatCACGTGgcaatttct
	N-box	–1526 to –1521	ggggagatCACGAGatgaatct
	N-box	–180 to –175	tgagctgcCACGCGggcccacc
*OsDREB1C*	G-box	–2095 to –2090	tggatggtCACGTGgcaggaaa
	G-box	–1439 to –1434	tactccctCACGTGcggctgga
	G-box	–473 to –303	gccatctcCACGTGgccacccc
	G-box	–181 to –176	tctcccgcCACGTGcgcgccgc
*OsDREB1E*	PBE	–674 to –668	ggagtgtgCACATGaagctcgt
	N-box	–552 to –547	caacgaatCACGCGctctccaa
	N-box	–534 to –529	ccaactcaCACGCGtccgcatc
	N-box	–397 to –392	cagagaagCACGAGcccaagcc
*OsDREB1F*	G-box	–291 to –296	cacgggcgCACGTGtttcatcc
	G-box	–260 to –254	tgaatcccCACGTGacgatcga
	N-box	–463 to –457	ttattttaCACGAGaaatttaa
*OsDREB1G*	PBE	–2769 to –2764	caaaaataCACATGtagaggtg
	N-box	–382 to –373	cgccgtccCACGAGacaacgag
	N-box	–91 to –86	tccctctcCACGCGctaaacta

*The N-box, PBE (PIF binding E-box), and G-box are marked by capital letters.*

### phyB Interacts with OsPIL16 *In vivo*

**FIGURE 6:**
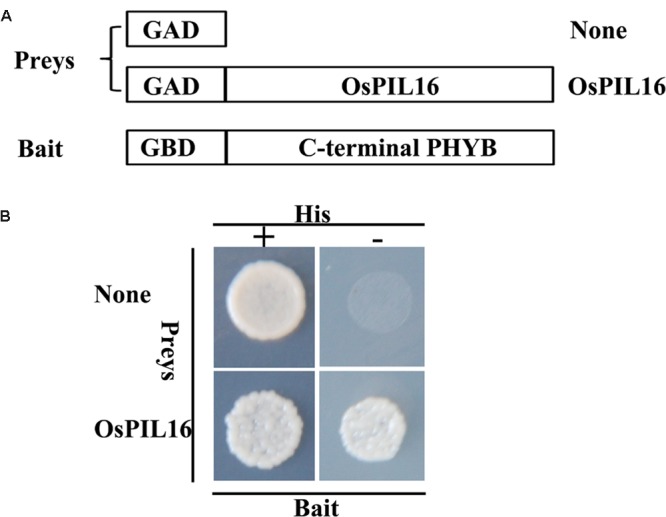
**Analysis of the interaction between OsPIF16 and OsPHYB using a yeast two-hybrid assay. (A)** Protein constructs used in the yeast two-hybrid assay. OsPIF16 was used as a prey in a translational fusion with the GAL4 AD. The C-terminal non-photoactive region of rice PHYB was fused with the GAL4 BD to use as a bait. **(B)** Analysis of protein–protein interactions in yeast growing in medium with (left) or without (right) histidine. The negative control was the interaction between OsPIF16 and GBD.

Recent evidence suggests that phytochrome signaling is initiated by the direct interaction of the biologically active forms of phytochromes with PIF members in *Arabidopsis* (Castillon et al., 2007). To examine the physical interaction between phyB and OsPIL16, we performed a yeast two-hybrid assay, using the coding region of *OsPIF16* as prey and the C-terminal non-photoactive coding region of *phyB* as bait. Three independent clones were used for each sample. As shown in **Figure 6** and Supplementary Figure 4, OsPIL16 interacted with the OsPHYB C-terminal domain. Thus, we speculated that phyB affects cold responses through an OsPIL16-mediated pathway.

## Discussion

**FIGURE 7:**
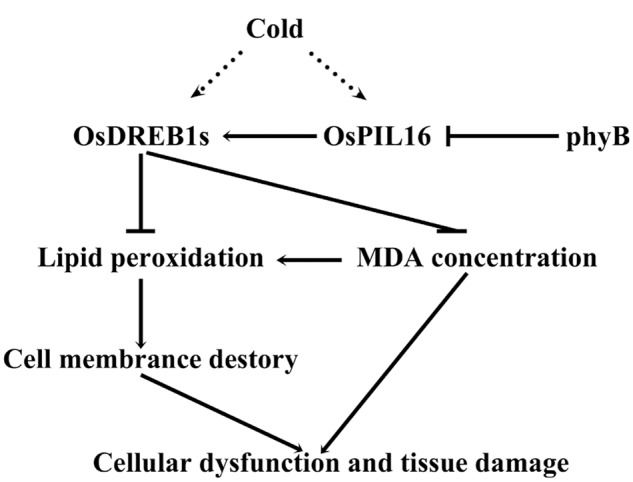
**Proposed model of the relationship between phytochrome B and cold stress.** Cold stress induces the expression of *OsDREB1*s. Phytochrome B negatively regulates *OsDREB1* genes by inhibiting the expression of *OsPIL16*. OsDREB1s inhibit lipid peroxidation to alleviate cold-induced membrane damage under cold stress. OsDREB1s also inhibit MDA accumulation to reduce cold damage and enhance cold tolerance. Arrows denote positive effects, bars indicate negative roles, the dotted line indicates indirect regulation, and solid lines indicate direct regulation.

phyB plays an important role in rice photomorphogenesis (Takano et al., 2005; Jumtee et al., 2009). In this study, we demonstrated that phyB deficiency resulted in improved cold tolerance compared with the WT though the regulation of *OsDREB1* gene expression via an OsPIL16-mediated pathway (**Figure 7**).

### The Enhanced Cold Tolerance of *phyB* Mutants Is Mainly Attributable to Increased Membrane Integrity and Reduced MDA Content

Cold stress in rice seedlings can negatively affect growth and development or even cause death (Zhang et al., 2014). In this study, phyB deficiency enhanced cold tolerance compared with the WT. One of the major forms of cold damage is severe membrane injury. ELI is an indicator of membrane integrity (Heidarvand and Maali-Amiri, 2013). The ELI was clearly decreased in the *phyB* mutants compared with the WT (**Figure 1C**), indicating that phyB deficiency increased membrane integrity under cold stress. This result is consistent with our previous report that a *phyB* mutant had a more stable chloroplast structure under cold stress (Yang et al., 2013). MDA is the degradation product of polyunsaturated lipids, and the increased membrane integrity of the *phyB* mutant can be partly attributed to reduced lipid peroxidation as shown by the reduced MDA content (**Figure 1D**). However, Yang et al. (2013) revealed that *phyB* mutants had higher unsaturated fatty acid contents in their membrane lipids, which also partly contributes to the increased membrane integrity of the *phyB* mutant (Yang et al., 2013). Therefore, we speculate that phyB deficiency improves membrane integrity in rice, which is an important factor for enhanced cold tolerance in *phyB* mutants. Additionally, MDA is a reactive aldehyde that initiates toxic stress in cells and subsequently causes cellular dysfunction and tissue damage (Zhang et al., 2014). Thus, we speculate that the reduced MDA content under cold stress caused by phyB deficiency is likely one of the factors contributing to cold tolerance in the *phyB* mutants.

### phyB Regulates *OsDREB1* Expression through OsPIFs to Affect Rice Cold Tolerance

C repeat binding factor/DREB1s play an important role in cold signal transduction (Cook et al., 2004; Zhao et al., 2016). Overexpression of rice or *Arabidopsis DREB1*s can enhance cold tolerance (Jagloottosen et al., 1998; Dubouzet et al., 2003; Zhang et al., 2009). Interestingly, we found that the *phyB* mutant had higher expression levels of *OsDREB1*s (**Figure 2**), which is probably related to the enhanced cold tolerance in the *phyB* mutant. Phytochromes have been reported to regulate *DREB1* gene expression in *Arabidopsis*. One study identified phyB as a positive regulator of cold-inducible DRE-controlled expression (Kim et al., 2002). However, the results of a subsequent study indicated a repressive role for phyB and phyD in the control of the CBF-responsive regulon (Franklin and Whitelam, 2007). Kidokoro et al. (2009) also showed that phyB-mediated light signaling had a repressive effect on *DREB1* gene expression in *Arabidopsis*, consistent with our results in this study (Kidokoro et al., 2009). Xu et al. (2011) found that overexpression of *ZmCBF3* in rice reduced the MDA concentration (Xu et al., 2011). In this context, we speculate that the reduced MDA concentration in the *phyB* mutant probably contributed to the upregulation of *OsDREB1* genes in our study.

How does phytochrome-mediated pathway regulate *DREB1* family gene expression? PIFs, as central players in phytochrome-mediated light signaling networks, have been reported to regulate *DREB1* gene expression in *Arabidopsis*. PIF7 can bind to the promoter of *DREB1C*, and the *pif7* null mutant was shown to have altered transcript levels of *DREB1B* and *DREB1C* (Kidokoro et al., 2009). PIF4 can downregulate the CBF pathway and freezing tolerance under LD conditions (Lee and Thomashow, 2012). There are six OsPIFs (*OsPIL11*–*OsPIL16*) in rice (Nakamura et al., 2007). The functions of OsPIL13 and OsPIL15 in rice growth and development have been reported. Overexpression of *OsPIL13* in transgenic rice plants promoted internode elongation (Todaka et al., 2012). OsPIL15 represses seedling growth in the dark, and when exposed to R or FR light, *OsPIL15*-OX lines can relieve growth retardation and promote seedling elongation (Zhou et al., 2014). However, to our knowledge, the roles of OsPIL13 and OsPIL15 in regulating *OsDREB1* gene expression have not been reported. In this study, our results showed that OsPIL16 upregulated the expression of multiple *OsDREB1* genes (**Figure 4E**), which is consistent with the enhanced cold tolerance of *OsPIL16*-OX transgenic lines (**Figure 4D**). OsPIL14 was reported to downregulate *OsDREB1B* gene expression in rice protoplasts by binding to the N-box motif of the *OsDREB1B* promoter (Cordeiro et al., 2016). How does OsPIL16, as a bHLH transcription factor, regulate the expression of *DREB1*s? bHLH proteins are able to bind to hexameric E-box (CANNTG) or N-box motifs, depending on their transcriptional activity (Cordeiro et al., 2016). A previous study revealed that PIFs not only bind to the G-box *in vitro* and *in vivo*, and also bind weakly *in vitro* to PBE (Kim et al., 2008, 2016; Zhang et al., 2013; Pfeiffer et al., 2014). We found that there were N-boxes, PBE or G-boxes in the promoter regions of the *OsDREB1* genes (**Table 1**). ChIP-qPCR assay confirmed that OsPIL16 can bind to the N-box of *OsDREB1B* promoter (Supplementary Figure 3), thus to regulate the expression of *OsDREB1B*.

To dissect the relationship between phyB and OsPILs, we compared the transcript levels of OsPILs in the WT and *phyB* mutant. The transcript levels of both *OsPIL15* and *OsPIL16* were significantly higher in the *phyB* mutant than in the WT (**Figure 2**). However, *OsPIL15*-OX did not show higher *OsDREB1* transcript levels (Supplementary Figure 2), which is probably consistent with the similar cold response of the *OsPIL15*-OX line to the WT. Unlike *OsPIL15*-OX, *OsPIL16*-OX had higher *OsDREB1* transcript levels (**Figure 4D**), which contributed to the enhanced cold tolerance in the *OsPIL16*-OX lines (**Figure 4C**). To explain how phyB affects *OsDREB1s* family gene expression through OsPIL16, it is necessary to clarify the relationship between phyB and OsPIL16. Therefore, we examined the regulation of phyB on *OsPIL16* transcript levels and physical interaction between phyB and OsPIL16 proteins. As shown in the results (**Figure 3**; Supplementary Figure 1), phyB perceives the R light to inhibit *OsPIL16* expression at the transcriptional level. Physical interaction between OsPIL16 and OsPHYB were also confirmed by Y2H (**Figure 6**; Supplementary Figure 4). There is a well-known model of PIF function in phytochrome signaling pathways in *Arabidopsis* (Castillon et al., 2007; Leivar and Quail, 2011). Light signals induce photoconversion of phytochromes to the active Pfr forms before nuclear migration. In the nucleus, phytochromes physically interact with PIFs, which results in phosphorylation of PIFs. The phosphorylated forms of PIFs are subsequently degraded by the 26S proteasome. The light-induced proteolytic removal of PIFs results in relieving the negative regulation of photomorphogenesis. However, Park et al. (2012) reported that phyB inhibits the regulatory activity of PIF1 and PIF3 by two different modes of action: by releasing them from their target promoters and by mediating their degradation. In this study, OsPIL16 induced *OsDREB1s* gene expression probably through binding to the N-box of their promoters. Thus, OsPIL16 is the positive regulator of *OsDREB1* gene expression and rice cold tolerance. However, the physical interaction between phyB and OsPIL16 proteins inhibits the *OsDREB1* gene expression by either degradation of OsPIL16 protein or by inhibiting the bind of OsPIL16 to N-box of *OsDREB1* gene promoter. This hypothesis can explain the higher transcript levels of *OsDREB1s* in the *phyB* mutants than in the WT.

In summary, we determined the function of phyB in the cold tolerance of rice using genetic and physiological approaches. Although our findings suggest that phyB affect rice cold tolerance through OsPIL16 pathway in this study, some other pathways, for example ABA pathway and ICE pathway, are probably also involved in the phyB-regulated cold responses in rice. ABA has been shown to increase levels of CBF1 transcript and protein (Knight et al., 2004). Our previous result revealed that light signals mediated by phytochrome B affect ABA pathway in rice (Gu et al., 2012). To test this possibility, we must produce the null mutants of *pil16* and *phyBpil16* double mutant in the future experiments. In addition, further work is needed to decipher the mechanisms by which OsPIL16 regulates OsDREB1s.

## Author Contributions

YH and XX conceived the idea, led the study design, data analysis, and manuscript writing. YL performed southern blot, assisted in quantitative PCR and yeast two-hybrid. CZ and LX performed vector construction and rice transformation. LC and GZ assisted in the measurement of ELI and MDA. LC and YH performed ChIP-qPCR. JZ assisted in rice growing.

## Conflict of Interest Statement

The authors declare that the research was conducted in the absence of any commercial or financial relationships that could be construed as a potential conflict of interest.

## Footnotes

1 http://rice.plantbiology.msu.edu/

## Abbreviations

bHLH: helix-loop-helix
CBF: C repeat binding factor
ChIP: chromatin immunoprecipitation
COR: COLD REGULATED
d: day
DREB: dehydration-responsive element binding protein
ELI: electrolyte leakage index
FR: far-red
IgG: immunoglobulin G
MDA: malondialdehyde
OX: overexpression
PBE: PIF binding E-box
phyB: phytochrome B
PIF: phytochrome-interacting factor
PIL: phytochrome-interacting factor-like
R: red
WT: wild type

## References

[B1] Carvalho R. F. Campos M. L. Azevedo R. A. (2011). The role of phytochrome in stress tolerance. *J. Integr. Plant Biol.* 53 920–929. 10.1111/j.1744-7909.2011.01081.x 22040287

[B2] Castillon A. Shen H. Huq E. (2007). Phytochrome Interacting Factors: central players in phytochrome-mediated light signaling networks. *Trends Plant Sci.* 12 514–521. 10.1016/j.tplants.2007.10.001 17933576

[B3] Cook D. Fowler S. Fiehn O. Thomashow M. F. (2004). A prominent role for the CBF cold response pathway in configuring the low-temperature metabolome of *Arabidopsis*. *Proc. Natl. Acad. Sci. U.S.A.* 101 15243–15248. 10.1073/pnas.0406069101 15383661 PMC524070

[B4] Cordeiro A. M. Figueiredo D. D. Tepperman J. Borba A. R. Lourenco T. Abreu I. A. (2016). Rice phytochrome-interacting factor protein OsPIF14 represses OsDREB1B gene expression through an extended N-box and interacts preferentially with the active form of phytochrome B. *Biochim. Biophys. Acta* 1859 393–404. 10.1016/j.bbagrm.2015.12.008 26732823 PMC4824199

[B5] Dubouzet J. G. Sakuma Y. Ito Y. Kasuga M. Dubouzet E. G. Miura S. (2003). OsDREB genes in rice, *Oryza sativa* L., encode transcription activators that function in drought-, high-salt- and cold-responsive gene expression. *Plant J.* 33 751–763. 10.1046/j.1365-313X.2003.01661.x 12609047

[B6] Franklin K. A. Quail P. H. (2010). Phytochrome functions in *Arabidopsis* development. *J. Exp. Bot.* 61 11–24. 10.1093/jxb/erp304 19815685 PMC2800801

[B7] Franklin K. A. Whitelam G. C. (2007). Light-quality regulation of freezing tolerance in *Arabidopsis thaliana*. *Nat. Genet.* 39 1410–1413. 10.1038/ng.2007.3 17965713

[B8] Gilmour S. J. Fowler S. G. Thomashow M. F. (2004). *Arabidopsis* transcriptional activators CBF1, CBF2, and CBF3 have matching functional activities. *Plant Mol. Biol.* 54 767–781. 10.1023/B:PLAN.0000040902.06881.d4 15356394

[B9] Gu J. W. Liu J. Xue Y. J. Zang X. Xie X. Z. (2011). Functions of phytochrome in rice growth and development. *Rice Sci.* 18 231–237. 10.1016/S1672-6308(11)60032-2

[B10] Gu J. W. Zhang F. Zhao J. Zhou J. J. Qian F. Q. Yan L. H. (2012). Light signals mediated by phytochrome B affect abscisic acid pathway in rice (in Chinese). *Chin. Sci. Bull.* 57 2371–2379. 10.1360/972011-2561

[B11] Heidarvand L. Amiri R. M. (2010). What happens in plant molecular responses to cold stress? *Acta Physiol. Plant.* 32 419–431. 10.1007/s11738-009-0451-8

[B12] Heidarvand L. Maali-Amiri R. (2013). Physio-biochemical and proteome analysis of chickpea in early phases of cold stress. *J. Plant Physiol.* 170 459–469. 10.1016/j.jplph.2012.11.021 23395538

[B13] Hiei Y. Ohta S. Komari T. Kumashiro T. (1994). Efficient transformation of rice (*Oryza sativa* L.) mediated by *Agrobacterium* and sequence analysis of the boundaries of the T-DNA. *Plant J.* 6 271–282. 10.1046/j.1365-313X.1994.6020271.x 7920717

[B14] Hood E. E. Gelvin S. B. Melchers L. S. Hoekema A. (1993). New *Agrobacterium* helper plasmid for gene transfer to plants (EHA105). *Transgenic Res.* 2 208–218. 10.1007/BF01977351

[B15] Ito Y. Katsura K. Maruyama K. Taji T. Kobayashi M. Seki M. (2006). Functional analysis of rice DREB1/CBF-type transcription factors involved in cold-responsive gene expression in transgenic rice. *Plant Cell Physiol.* 47 141–153. 10.1093/pcp/pci230 16284406

[B16] Jagloottosen K. R. Gilmour S. J. Zarka D. G. Schabenberger O. Thomashow M. F. (1998). *Arabidopsis* CBF1 overexpression induces COR genes and enhances freezing tolerance. *Science* 280 104–106. 10.1126/science.280.5360.104 9525853

[B17] Jeong J. Choi G. (2013). Phytochrome-interacting factors have both shared and distinct biological roles. *Mol. Cells* 35 371–380. 10.1007/s10059-013-0135-5 23708772 PMC3887866

[B18] Jumtee K. Okazawa A. Harada K. Fukusaki E. Takano M. Kobayashi A. (2009). Comprehensive metabolite profiling of phyA phyB phyC triple mutants to reveal their associated metabolic phenotype in rice leaves. *J. Biosci. Bioeng.* 108 151–159. 10.1016/j.jbiosc.2009.03.010 19619864

[B19] Kidokoro S. Maruyama K. Nakashima K. Imura Y. Narusaka Y. Shinwari Z. K. (2009). The phytochrome-interacting factor PIF7 negatively regulates DREB1 expression under circadian control in *Arabidopsis*. *Plant Physiol.* 151 2046–2057. 10.1104/pp.109.147033 19837816 PMC2785984

[B20] Kim D. H. Yamaguchi S. Lim S. Oh E. Park J. Hanada A. (2008). SOMNUS, a CCCH-type zinc finger protein in *Arabidopsis*, negatively regulates light-dependent seed germination downstream of PIL5. *Plant Cell* 20 1260–1277. 10.1105/tpc.108.058859 18487351 PMC2438461

[B21] Kim H. J. Kim Y. K. Park J. Y. Kim J. (2002). Light signalling mediated by phytochrome plays an important role in cold-induced gene expression through the C-repeat/dehydration responsive element (C/DRE) in *Arabidopsis thaliana*. *Plant J.* 29 693–704. 10.1046/j.1365-313X.2002.01249.x 12148528

[B22] Kim J. Kang H. Park J. Kim W. Yoo J. Lee N. (2016). PIF1-interacting transcription factors and their binding sequence elements determine the in vivo targeting sites of PIF1. *Plant Cell* 28 1388–1405. 10.1105/tpc.16.00125 27303023 PMC4944412

[B23] Kim S.-I. Tai T. H. (2011). Evaluation of seedling cold tolerance in rice cultivars: a comparison of visual ratings and quantitative indicators of physiological changes. *Euphytica* 178 437–447. 10.1007/s10681-010-0343-4

[B24] Knight H. Zarka D. G. Okamoto H. Thomashow M. F. Knight M. R. (2004). Abscisic acid induces *CBF* gene transcription and subsequent induction of cold-regulated genes via the CRT promoter element. *Plant Physiol.* 135 1710–1717. 10.1104/pp.104.043562 15247382 PMC519084

[B25] Lee C. M. Thomashow M. F. (2012). Photoperiodic regulation of the C-repeat binding factor (CBF) cold acclimation pathway and freezing tolerance in *Arabidopsis thaliana*. *Proc. Natl. Acad. Sci. U.S.A.* 109 15054–15059. 10.1073/pnas.1211295109 22927419 PMC3443188

[B26] Leivar P. Quail P. H. (2011). PIFs: pivotal components in a cellular signaling hub. *Trends Plant Sci.* 16 19–28. 10.1016/j.tplants.2010.08.003 20833098 PMC3019249

[B27] Li L. Ljung K. Breton G. Schmitz R. J. Pruneda-Paz J. Cowing-Zitron C. (2012). Linking photoreceptor excitation to changes in plant architecture. *Genes Dev.* 26 785–790. 10.1101/gad.187849.112 22508725 PMC3337452

[B28] Liu D. Liu Y. Rao J. Wang G. Li H. Ge F. (2013). Overexpression of the glutathione S-transferase gene from *Pyrus pyrifolia* fruit improves tolerance to abiotic stress in transgenic tobacco plants. *Mol. Biol.* 47 515–523. 10.1134/S0026893313040109 24466748

[B29] Luo Q. Lian H. L. He S. B. Li L. Jia K. P. Yang H. Q. (2014). COP1 and phyB physically interact with PIL1 to regulate its stability and photomorphogenic development in *Arabidopsis*. *Plant Cell* 26 2441–2456. 10.1105/tpc.113.121657 24951480 PMC4114944

[B30] Mao D. Chen C. (2012). Colinearity and similar expression pattern of rice DREB1s reveal their functional conservation in the cold-responsive pathway. *PLoS ONE* 7:e47275 10.1371/journal.pone.0047275 PMC3473061 23077584

[B31] Murray M. G. Thompson W. F. (1980). Rapid isolation of high molecular weight plant DNA. *Nucleic Acids Res.* 8 4321–4325. 10.1093/nar/8.19.4321 7433111 PMC324241

[B32] Nagatani A. (2010). Phytochrome: structural basis for its functions. *Curr. Opin. Plant Biol.* 13 565–570. 10.1016/j.pbi.2010.07.002 20801708

[B33] Nakamura Y. Kato T. Yamashino T. Murakami M. Mizuno T. (2007). Characterization of a set of phytochrome-interacting factor-like bHLH proteins in *Oryza sativa*. *Biosci. Biotechnol. Biochem.* 71 1183–1191. 10.1271/bbb.60643 17485859

[B34] Park E. Park J. Kim J. Nagatani A. Lagarias J. C. Choi G. (2012). Phytochrome B inhibits binding of phytochrome-interacting factors to their target promoters. *Plant J.* 72 537–546. 10.1111/j.1365-313X.2012.05114.x 22849408 PMC3489987

[B35] Pfeiffer A. Shi H. Tepperman J. M. Zhang Y. Quail P. H. (2014). Combinatorial complexity in a transcriptionally centered signaling hub in *Arabidopsis*. *Mol. Plant* 7 1598–1618. 10.1093/mp/ssu087 25122696 PMC4587546

[B36] Rockwell N. C. Lagarias J. C. (2006). The structure of phytochrome: a picture is worth a thousand spectra. *Plant Cell* 18 4–14. 10.1105/tpc.105.038513 16387836 PMC1323480

[B37] Sutinen M.-L. Palta J. P. Reich P. B. (1992). Seasonal differences in freezing stress resistance of needles of *Pinus nigra* and *Pinus resinosa*: evaluation of the electrolyte leakage method. *Tree Physiol.* 11 241–254. 10.1093/treephys/11.3.241 14969949

[B38] Takano M. Inagaki N. Xie X. Yuzurihara N. Hihara F. Ishizuka T. (2005). Distinct and cooperative functions of phytochromes A, B, and C in the control of deetiolation and flowering in rice. *Plant Cell* 17 3311–3325. 10.1105/tpc.105.035899 16278346 PMC1315371

[B39] Takano M. Kanegae H. Shinomura T. Miyao A. Hirochika H. Furuya M. (2001). Isolation and characterization of rice phytochrome A mutants. *Plant Cell* 13 521–534. 10.1105/tpc.13.3.521 11251094 PMC135516

[B40] Thomashow M. F. (2001). So what’s new in the field of plant cold acclimation? Lots! *Plant Physiol.* 125 89–93. 10.1104/pp.125.1.89 11154304 PMC1539333

[B41] Todaka D. Nakashima K. Maruyama K. Kidokoro S. Osakabe Y. Ito Y. (2012). Rice phytochrome-interacting factor-like protein OsPIL1 functions as a key regulator of internode elongation and induces a morphological response to drought stress. *Proc. Natl. Acad. Sci. U.S.A.* 109 15947–15952. 10.1073/pnas.1207324109 22984180 PMC3465374

[B42] Wang F. Guo Z. Li H. Wang M. Onac E. Zhou J. (2016). Phytochrome A and B function antagonistically to regulate cold tolerance via abscisic acid-dependent jasmonate signaling. *Plant Physiol.* 170 459–471. 10.1104/pp.15.01171 26527654 PMC4704577

[B43] Wang Q. Guan Y. Wu Y. Chen H. Chen F. Chu C. (2008). Overexpression of a rice OsDREB1F gene increases salt, drought, and low temperature tolerance in both *Arabidopsis* and rice. *Plant Mol. Biol.* 67 589–602. 10.1007/s11103-008-9340-6 18470484

[B44] Williams B. J. Pellett N. E. Klein R. M. (1972). Phytochrome control of growth cessation and initiation of cold acclimation in selected woody plants. *Plant Physiol.* 50 262–265. 10.1104/pp.50.2.262 16658153 PMC366121

[B45] Xu M. Li L. Fan Y. Wan J. Wang L. (2011). ZmCBF3 overexpression improves tolerance to abiotic stress in transgenic rice (*Oryza sativa*) without yield penalty. *Plant Cell Rep.* 30 1949–1957. 10.1007/s00299-011-1103-1 21811828

[B46] Yang J. C. Li M. Xie X. Z. Han G. L. Sui N. Wang B. S. (2013). Deficiency of phytochrome B alleviates chilling-induced photoinhibition in rice. *Am. J. Bot.* 100 1860–1870. 10.3732/ajb.1200574 24018854

[B47] Zhang Q. Chen Q. Wang S. Hong Y. Wang Z. (2014). Rice and cold stress: methods for its evaluation and summary of cold tolerance-related quantitative trait loci. *Rice (N. Y.)* 7:24 10.1186/s12284-014-0024-3 PMC4182278 25279026

[B48] Zhang Y. Chen C. Jin X. F. Xiong A. S. Peng R. H. Hong Y. H. (2009). Expression of a rice DREB1 gene, OsDREB1D, enhances cold and high-salt tolerance in transgenic *Arabidopsis*. *BMB Rep.* 42 486–492. 10.5483/BMBRep.2009.42.8.486 19712584

[B49] Zhang Y. Mayba O. Pfeiffer A. Shi H. Tepperman J. M. Speed T. P. (2013). A quartet of PIF bHLH factors provides a transcriptionally centered signaling hub that regulates seedling morphogenesis through differential expression-patterning of shared target genes in *Arabidopsis*. *PLoS Genet.* 9:e1003244 10.1371/journal.pgen.1003244 PMC3561105 23382695

[B50] Zhao C. Zhang Z. Xie S. Si T. Li Y. Zhu J. K. (2016). Mutational evidence for the critical role of CBF genes in cold acclimation in *Arabidopsis*. *Plant Physiol.* 171 2744–2759. 10.1104/pp.16.00533 27252305 PMC4972280

[B51] Zhou J. Liu Q. Zhang F. Wang Y. Zhang S. Cheng H. (2014). Overexpression of OsPIL15, a phytochrome-interacting factor-like protein gene, represses etiolated seedling growth in rice. *J. Integr. Plant Biol.* 56 373–387. 10.1111/jipb.12137 24279300

[B52] Zhou J. Wang F. Deng P. Jing W. Zhang W. (2013). Characterization and mapping of a salt-sensitive mutant in rice (*Oryza sativa* L.). *J. Integr. Plant Biol.* 55 504–513. 10.1111/jipb.12048 23480486

