# Supplementary material for: Phytochrome B Negatively Affects Cold Tolerance by Regulating OsDREB1 Gene Expression through Phytochrome Interacting Factor-Like Protein OsPIL16 in Rice
Source: Front Plant Sci. 2016 Dec 26;7:1963. doi: 10.3389/fpls.2016.01963 (PMC5183628; doi:10.3389/fpls.2016.01963)
Supplement: Supplementary file 1 [file Table_1.DOCX]

Supplementary Material

Article Title

Yanan He, Yaping Li, Lixin Cui, Lixia Xie, Chongke Zheng, Guanhua Zhou, Jinjun Zhou, Xianzhi Xie*

*** Correspondence:** Xianzhi Xie: xzhxie2010@163.com

Supplementary Table 1. The primers used in this study.

| Primer name | Primer sequence (5＇-3＇) |
| --- | --- |
| OsEF-qF | TTTCACTCTTGGTGTGAAGCAGAT |
| OsEF-qR | GACTTCCTTCACGATTTCATCGTAA |
| OsDREB1A-qF | ACCTGTACTACGCGAGCTTG |
| OsDREB1A-qR | TAGTAGCTCCAGAGTGGGAC |
| OsDREB1B-qF | GGCAATGAGACTGAGGATGAA |
| OsDREB1B-qR | TCAGATGGAATCACAAAAGGAG |
| OsDREB1C-qF | TCGACCAGCCGTCCTACTAC |
| OsDREB1C-qR | CCAACTCGCGATCATCAGTA |
| OsDREB1D-qF | CAAAGCTTATCAGCAGTAGC |
| OsDREB1D-qR | GGTTAGTAGCAGAAAGACTTG |
| OsDREB1E-qF | GAATTCGAAATGCAGGGGTA |
| OsDREB1E-qR | CTCGCAGTCGTAGTCCTCCT |
| OsDREB1F-qF | AGGACGCCATCTTCGACAT |
| OsDREB1F-qR | GTCGAGAGATCTCCCAATCG |
| OsDREB1G-qF | CCCGTACTACGAGGTCATGG |
| OsDREB1G-qR | GCTACCTACGGCAGGATCAC |
| OsDREB1I-qF | GAGCCTGTACTACGCGAGCTTA |
| OsDREB1I-qR | TCAGCGATGTCGCTTGAGTC |
| OsDREB1J-qF | GTCACCCAACGACGACAAT |
| OsDREB1J-qR | CAAGCTCGCGAAGTACAGAT |
| OsPIL11-qF | CAACTAgCATCTCCTCCTCTACTT g |
| OsPIL11-qR | CTCTTTCTCTTTgAgCTgAgATgAC |
| OsPIL12-qF | gCCAggAACTAgCTgTgATg |
| OsPIL12-qR | CCA gACCAgCCTTATgCTTTg |
| OsPIL13-qF | CgAAgCAAATCgTgTAgAgA |
| OsPIL13-qR | CACAgAgTCAggTCAggTCA |
| OsPIL14-qF | ATgACTACCgggATTgTg |
| OsPIL14-qR | CTgATTCAggCCTTgAgC |
| OsPIL15-qF | gggATTggggTACgggATg |
| OsPIL15-qR | ATTgggCAgggAAAgTgA g |
| OsPIL16-qF | CgACTACCTCTTCATCAAgAATC |
| OsPIL16-qR | gACAgggTggTAgATgCAACCA |
| OsPIL16-F | AAGGATCCATGCTACGCGGGAACGACACC |
| OsPIL16-R | AACTAGTTCACGCCTGCTTCACGGCGGGG |
| OsPIL16-AD-F | AACATATGCTACGCGGGAACGACACCG |
| OsPIL16-AD-R | ATGGATCCTCACGCCTGCTTCACGGCGG |
| C-phyB-F1 | AACCATGGCAGAGGGCACAAGTAAC |
| C-phyB-R1 | ATCTGCAGCTTGTCCCCCTACTTG |
| OsDREB1B-G-F1 | TGGCCGTGGATATCAATCAC |
| OsDREB1B-G-R1 | CCTAGGTTAGGATAGCCATGTATTC |
| OsDREB1B-N-F1 | CTGAAGACAGCGAGAGTGTG |
| OsDREB1B-N-R1 | GGTAAGTGGTGGTCGTTACTC |
| OsPIL16-HA-R1 | AACTAGTCGCCTGCTTCACGGCGGGG |

**Supplementary Figure 1.** Effect of Red light on expression of *OsPIL16* in the WT and *phyB* mutant. Seeds from WT and *phyB* were de-husked, surface-sterilized, and grown in 0.4% agar were grown for 7 days at 28 °C in darkness, and then transferred to red linght for another 24 hours.

**Supplementary Figure 2.** *OsDREB1* expression levels in the *OsPIL15*-OX lines and WT. Real-time PCR was performed using cDNA derived from the leaves of three-leaf stage seedlings. Error bars indicate SE (*n* = 3).

**Supplementary Figure 3.** Western bolt analysis of *OsPIL16*-*HA-*OX line and WT. Seedlings of two weeks old WT and *OsPIL16*-*HA*-OX line were detected using anti-HA antibody.

**Supplementary Figure 4.** Analysis of the interaction between OsPIF16 and OsPHYB using a yeast two-hybrid assay. (A) Protein constructs used in the yeast two-hybrid assay. OsPIF16 was used as a prey in a translational fusion with the GAL4 AD. The C-terminal non-photoactive region of rice PHYB was fused with the GAL4 BD to use as a bait. (B) Analysis of protein–protein interactions in yeast growing in medium with (left) or without (right) histidine. The negative control was the interaction between OsPIF16 and GBD.
