# Supplementary figures and images for: Phytochrome B Negatively Affects Cold Tolerance by Regulating OsDREB1 Gene Expression through Phytochrome Interacting Factor-Like Protein OsPIL16 in Rice
Source: Front Plant Sci. 2016 Dec 26;7:1963. doi: 10.3389/fpls.2016.01963 (PMC5183628; doi:10.3389/fpls.2016.01963)

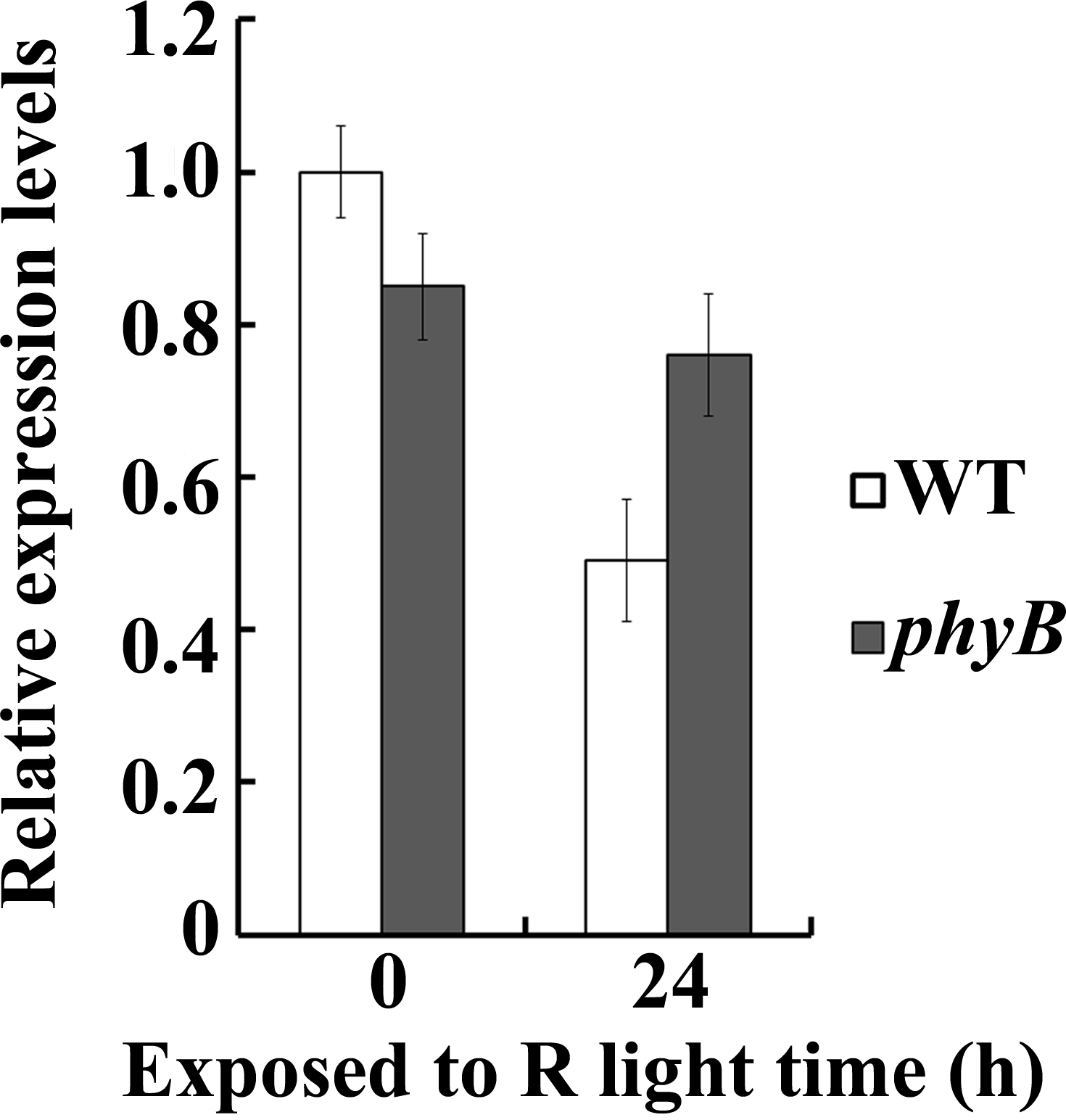

Supplement: Supplementary file 2 [file Image_1.TIF]

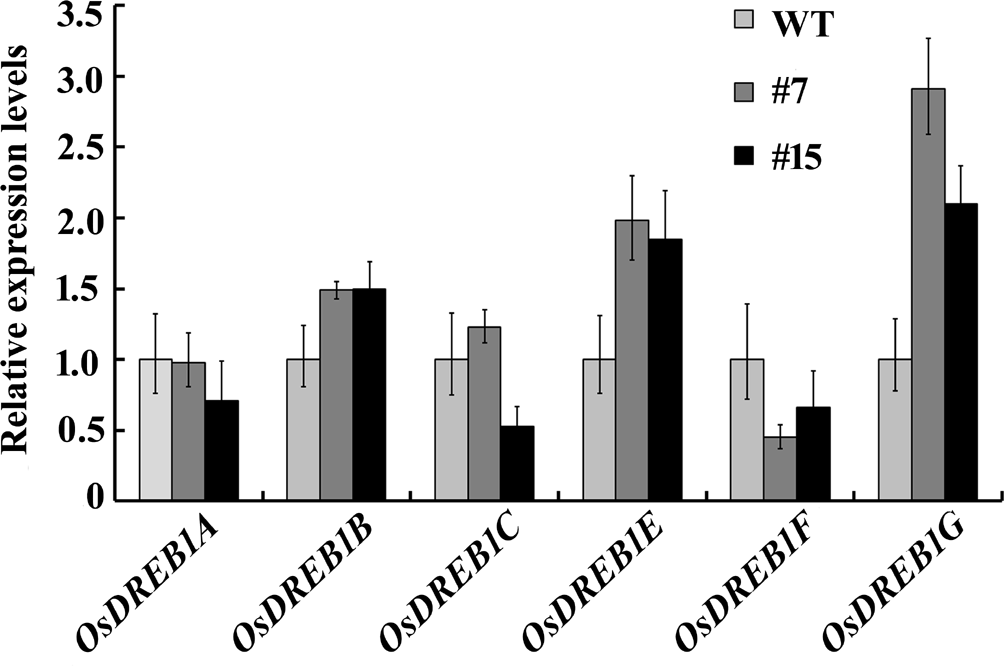

Supplement: Supplementary file 3 [file Image_2.TIF]

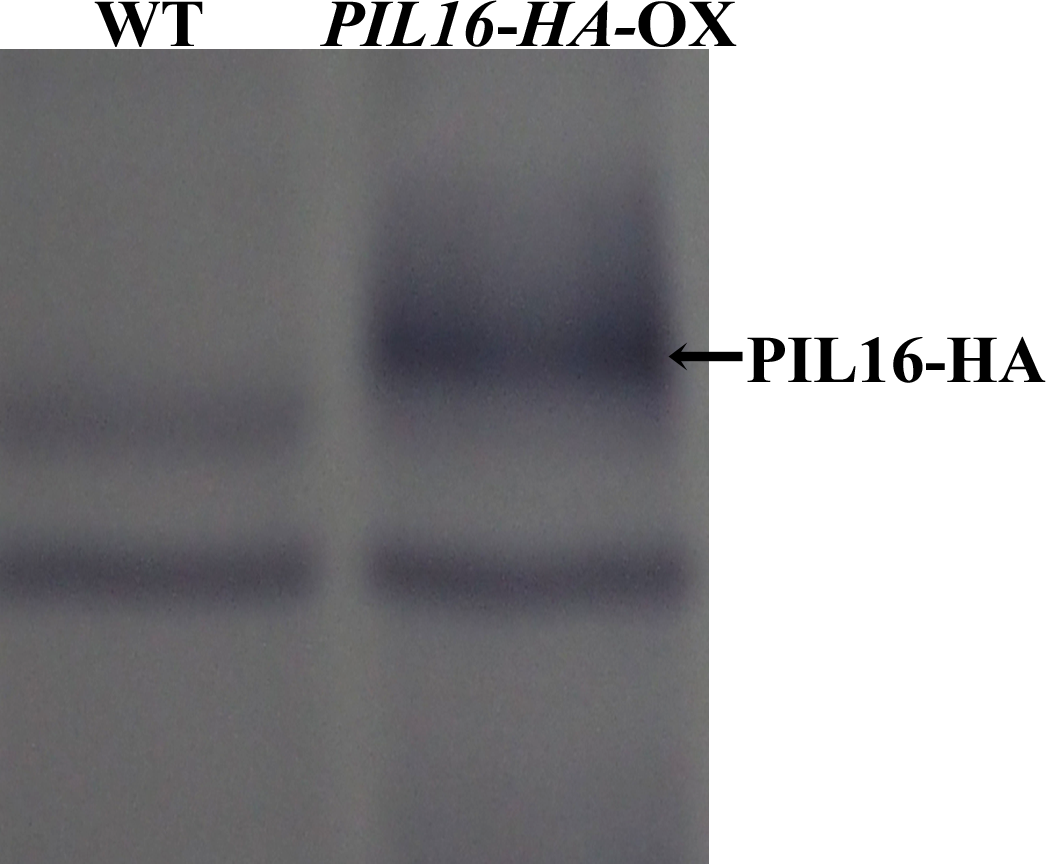

Supplement: Supplementary file 4 [file Image_3.TIF]

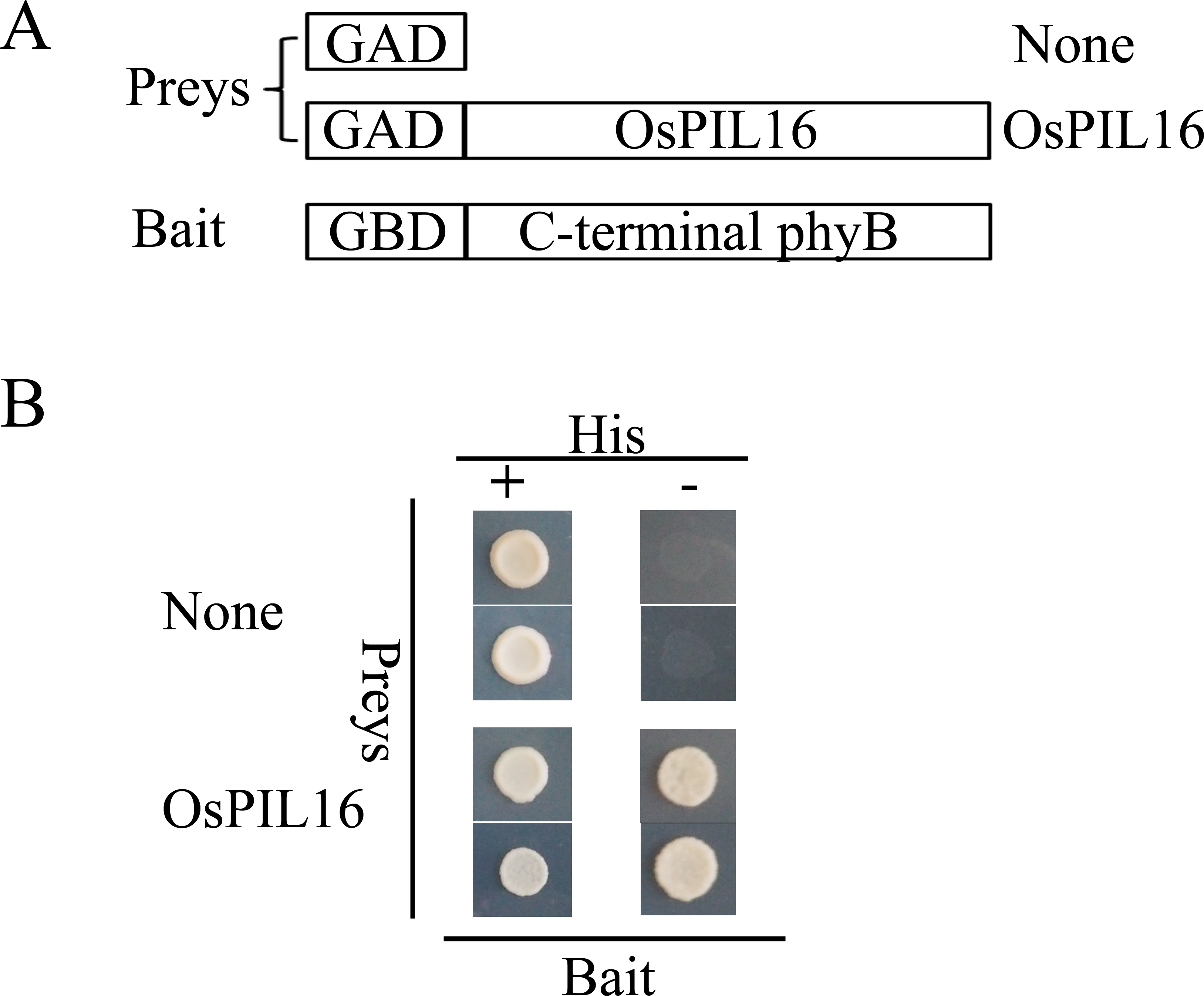

Supplement: Supplementary file 5 [file Image_4.TIF]
